# An integrative pan-cancer analysis revealing the difference in small ring finger family of SCF E3 ubiquitin ligases

**DOI:** 10.3389/fimmu.2022.968777

**Published:** 2022-08-18

**Authors:** Tingting Huang, Jiwei Li, Xinli Liu, Bingbing Shi, Shiqin Li, Han-Xiang An

**Affiliations:** ^1^Department of Medical Oncology, Xiang’an Hospital of Xiamen University, Xiamen, China; ^2^Department of Medical Oncology, Xiamen Key Laboratory of Endocrine-Related Cancer Precision Medicine, Xiamen, China; ^3^Department of Respiratory, Critical Care and Sleep Medicine, Xiang’an Hospital of Xiamen University, Xiamen, China; ^4^Department of Critical Care Medicine, The Affiliated Hospital of Putian University, Putian, China; ^5^Department of Gastroenterology, Xiamen Branch, Zhongshan Hospital, Fudan University, Xiamen, China

**Keywords:** pan-cancer, ring-finger proteins, SCF complex, prognosis, immune infiltration, difference

## Abstract

**Background:**

The SCF (Skp1-cullin-F-box proteins) complex is the largest family of E3 ubiquitin ligases that mediate multiple specific substrate proteins degradation. Two ring-finger family members RBX1/ROC1 and RBX2/RNF7/SAG are small molecular proteins necessary for ubiquitin ligation activity of the multimeric SCF complex. Accumulating evidence indicated the involvement of RBX proteins in the pathogenesis and development of cancers, but no research using pan-cancer analysis for evaluating their difference has been directed previously.

**Methods:**

We investigated RBX1/2 expression patterns and the association with clinicopathological features, and survivals of cancer patients obtained from the TCGA pan-cancer data. The binding energies of RBX1/2-CUL1 complexes were preliminarily calculated by using molecular dynamics simulations. Meanwhile, we assessed their immune infiltration level across numerous databases, including TISIDB and Timer database.

**Results:**

High expression levels of RBX1/2 were observed in most cancer types and correlated with poor prognosis of patients analyzed. Nonetheless, exceptions were observed: RBX2 expression in KICH was higher than normal renal tissues and played a detrimental role in KICH. The expression of RBX1 was not associated with the prognostic risk of KICH. Moreover, the combination of RBX1 and CUL1 expression is more stable than that of RBX2 and CUL1. RBX1/2 expression showed their own specific characteristics in tumor pathological stages and grades, copy number variation and immune components.

**Conclusions:**

These findings not only indicated that the difference of RBX1/2 might result in varying degrees of tumor progression, but also suggested that they might serve as biomarkers for immune infiltration in cancers, shedding new light on therapeutics of cancers.

## Introduction

The ubiquitin–proteasome system (UPS) is the major proteolytic system that degrades accumulated or misfolded proteins for cellular homeostasis (1, 2). It operates through the presentation of ubiquitin to the substrate proteins using a covalent modification pattern, which involves a series of multienzymes, i.e., Ubiquitin (Ub)-activating enzyme (E1), Ub-conjugating enzyme (E2) and Ub ligase (E3) (3). Among the three enzymes, the E3 ubiquitin ligases play a pivotal role in determining specificity of substrate proteolysis (4, 5). Based on the structural characteristics, E3 enzyme can be divided into four categories: RING E3s, HECT E3s, U-box E3s and RBR E3s (4). The SCF multisubunit complex, the most common RING E3s composing of a scaffold protein cullin1, a Ring protein (RBX1 or RBX2), an adaptor protein and a substrate receptor protein, is the largest family of E3s that promote the degradation of about 20% of UPS-regulated proteins (6, 7).

RBX1/2 usually ubiquitously expressed in human tissues, such as heart, colon, skeletal muscle, and testes (8, 9). RBX proteins can promote ubiquitin transfer from the E2 to the substrates and further enhances cullins activity, therefore, they constitute the catalytic cores of SCF complexes (10). Previous studies have confirmed that RBX proteins were found to be functionally non-redundant. Deletion of RBX1 in mice results in early embryo death (E7.5) due to proliferation failure in a wild-type RBX2 background, whereas inactivation of RBX2 causes late embryo death (E11.5-12.5) associated with cardiovascular defects (11, 12). Although both RBX proteins are highly conservative at protein level, share similar ring finger domain structure, their effect on the regulation of substrate degradation may vary (11). RBX1 mainly mediates proteolysis, including cell cycle regulators (e.g., cell cycle inhibitor p21/p27/p53/p57, and cyclin A/D/E), transcription factors (e.g., E2F1, FOXO1, myc, and c-Jun), DNA replication factor CDT1, and others. RBX2 promotes ubiquitination and degradation of a number of protein substrates, including c-Jun, DEPTOR, HIF-1α, IκBα, NF1, NOXA, p27 and procaspase-3, to degrade different substrates causing various phenotypes (13–16).

To be specific in cancers, RBX1 was shown to be highly expressed in bladder, gastric, prostate and renal cancer (17–19). Notably, RBX2, is rarely expressed in normal tissues, but highly expressed in lung, liver, gastric and renal cancer (20–22). Previous studies on the Ring finger family have focused on the oncogenic function and degradation ability of RBX1 and RBX2 in specific tumors, respectively, which provides a limited understanding of their role in SCF E3s. However, the difference of RBX members in pan-cancer has not been described. To explore the effect of RBX1/2 on the overall picture of SCF complex and in the tumor evolution, we comprehensively analyzed their difference in pan-cancer using the TCGA database in the present study. Their diversities were reflected in the following aspects including mRNA level, protein level, pathological features, prognosis and copy number variation, immune infiltration level.

## Materials and methods

### Evaluation the two cullin1-based complexes by binding free energy simulations

The CUL1-RBX1 and CUL1-RBX2 complexes were obtained from the Protein Data Bank (RCSB PDB www.rcsb.org) database (23). The molecular dynamic simulation for the CUL1-RBX1/2 complex used PDB ID: 1LDJ and 7 ONI as the templates. A molecular dynamic simulation was performed for the two complexes in a water environment (310 K temperature) with the force field charmm36-feb2021.ff using GROMACS software (24). The binding affinity was calculated using g_mmpbsa and the PyMOL software was used for visualization (25).

### The cancer genome atlas pan-cancer data

We used the UCSC Xena (https://xenabrowser.net/) to download TCGA pan-cancer data, including survival data, clinical data, stemness score (RNA based) and immune subtype (26). RBX1/2 expression was integrated by Perl software. We used the Wilcox test to assess the difference between normal and tumor tissues. P value less than 0.05 is considered as difference. A heatmap and box plot were illustrated by the R-package “ggpubr” and “pheatmap”, respectively. Furthermore, Correlation analysis among Ring finger family genes was performed by R-package “corrplot”.

### Clinicopathologic features and survival analysis of expression of Ring finger members

UALCAN was used to analyze the RBX-proteins expression in several cancers, including BRAC, OV, UCEC and PAAD (27). ∗P< 0.05, ∗∗P< 0.01, and ∗∗∗P< 0.001. Additionally, we obtained box plots of the RBX1/2 expression in different pathological grades and stages *via* the TISIDB database (28) (http://cis.hku.hk/TISIDB/index.php). Survival analysis of RBX1/2 was used for the “survival” and “survminer” R package. A difference of p less than 0.05 was statistically significant. Meanwhile, we downloaded the TCGA pan-cancer mRNA expression and survival data to conduct the Cox analysis for illustrating the association between RBX1/2 expression and the survival of patients.

### RBX1/2 CNV profile in pan-cancer based on GSCA

Gene Set Cancer Analysis (GSCA) platform is a web server that integrated multiomics data based on TCGA database (29) (http://bioinfo.life.hust.edu.cn/web/GSCA/). Based on CNV module, the proportion of RBX1/2 heterozygous/homozygous and amplification/deletion, Spearman correlation between RBX1/2 mRNA expression and CNV, and the survival difference between their CNV and wild type were displayed in pan-cancer.

### Correlation analysis of Ring finger family gene expression with immune components in pan-cancer

The correlation between Ring finger family expression and immune subtypes of different cancer types were explored *via* the TISIDB database. Furthermore, we selected four types of cancers (COAD, GBM, LIHC, LUAD) to analyze the relationship between RBX1/2 and immune infiltration using Timer database (30)(https://cistrome.shinyapps.io/timer/). Moreover, the associations of RBX1/2 levels with 47 common immune checkpoint genes selected were also evaluated. R software was used to calculate the correlation between RBX1/2 expression and TMB/MSI and the Fmsb R package was used for visualization. Then, we performed the tumor microenvironment analysis for obtaining the estimate score profile by using the “estimate” R package, and the Spearman correlation test for conducting the correlation analysis between RBX1/2 expression and immune score, estimate score, stromal score, DNAss, RNAss and tumor purity in pan-cancer.

### Cell culture

All human breast cancer cell lines (MDA-231, BT-474, MCF-7) and normal breast epithelial cell (MCF-10A), lung cancer cell lines (H1975, A549, PC9) and normal lung epithelial cell (BEAS-2B), colorectal cancer cell lines (HCT116, SW480, SW620) and normal colon epithelial cell (HCoEpic), renal cancer cell lines (Caki-1, 786-O, 769-P) and normal renal tubular epithelial cell (HK-2) were purchased from the American Type Tissue Collection (ATCC) and cultured according to the manufacturer’s instructions.

### qRT-PCR analysis

cDNA reverse transcription and fluorescence quantitative PCR amplification were performed using SPARKscript IISYBR Green qRT-PCR Kit (Shandong Sparkjade Biotechnology Co., Ltd.) as previously reported (31). The primers used were as follows: RBX1 forward, 5′-TTGTGGTTGATAACTGTGCCAT -3′,

RBX1 reverse, 5′-GACGCCTGGTTAGCTTGACAT -3′;
RBX2 forward, 5′-TGGAAGACGGAGAGGAAACCT -3′,
RBX2 reverse, 5′-TGAGGGAGAACATCTTGTCGC -3′
β-Actin forward, 5′- CGTGCGTGACATTAAGGAGAAG -3′,
β-Actin reverse, 5′- GGAAGGAAGGCTGGAAGAGTG -3′;.

All genes were normalized to β-actin, and the 2^−ΔΔCt^ method was applied to evaluate the relative levels of genes. The comparison between the experimental group and the normal group was performed using the Dunnett’s t test. P less than 0.05 was considered statistically significant.

## Results

### Molecular dynamics simulations and free energy calculations of the CUL1-RBX1 and CUL1-RBX2 complexes

The SCF complexes are Ring-type E3s that composited of cullin1, SKP1, RBX1/2 and a member of the F-box protein family. Although the abundance of SCF is increased by the variety of F-box proteins, they share the two ring components RBX1 and RBX2 (32–34). RBX1 is constitutively expressed and induced upon mitogen, whereas RBX2 is stress-inducible and induced upon UV, TPA or ROS (14). In this study, we separately calculated the binding affinity of CUL1-RBX1 and CUL1-RBX2 complexes to rough compare stability of SCF complex formed by RBX1/2. The binding energy calculated by the former was -262.59 kJ/mol and the latter was -146.8 kJ/mol (**Supplementary Figures 1A, B**). The result displayed the combination of RBX1 and CUL1 may be more stable than that of RBX2 and CUL1, suggesting that RBX1 is more likely to form stable SCF complexes to degrade more substrates.

### Expression of RBX1/2 in various types of cancers and association with pathological characteristics

**Figure 1:**
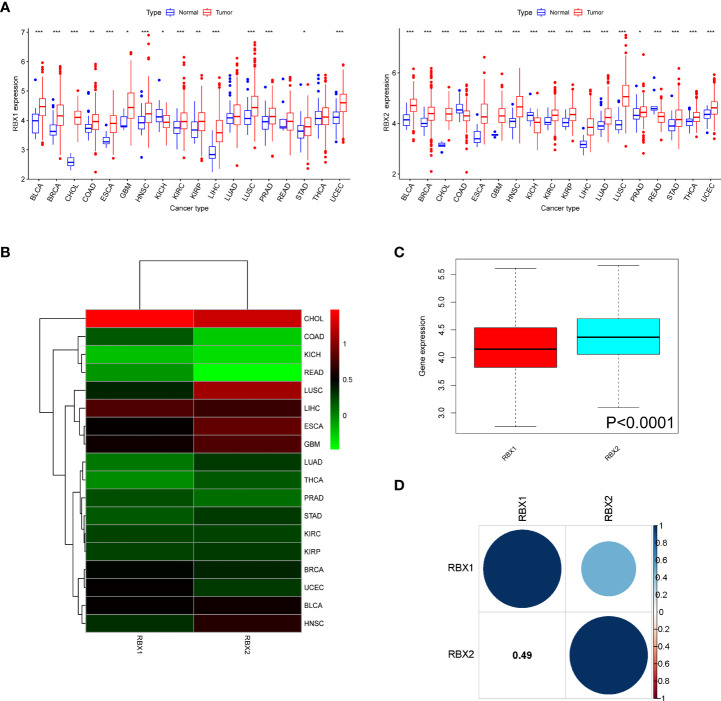
The mRNA expression patterns of RBX1/2 in cancers. **(A)** Comparison of RBX1/2 expression between tumor and normal samples. **(B)** Heatmap showing the difference of RBX1/2 gene expression in 18 cancer types from TCGA database. The red and green indicate the high or low expression, respectively. **(C)** Boxplot illustrating the distribution of RBX1/2 gene expression in various cancer. **(D)** The correlation between RBX1 and RBX2. The blue dot indicated the positive correlation. *P < 0.05, **P < 0.01, ***P < 0.001.

We performed a scale analysis of the expression of RBX1/2 from the TCGA database and found that they are highly expressed in most cancers. However, there were a few apparent exceptions in the 18 types of cancers, a lower RBX1 expression was detected in KICH compare to the matches normal tissues, whereas RBX2 was under expressed in COAD and READ in addition to KICH (**Figure 1A**). To validate the differences of RBX1/2 expression, we analysed transcriptional expression of these both genes in various tumor cell lines of four common types of cancer (breast, lung, colorectal and renal cancer) and normal cells. Except for the expression of RBX1 in lung cancer and RBX2 in COAD, the experimental results are basically consistent with the bioinformatics analysis (**Supplementary Figure 2**).

Further analysis revealed that there were significant difference of Ring finger genes expression comparing primary tumor to adjacent normal tissues, for example, RBX2 expression in COAD tissues was lower than adjacent non-COAD tissues, while RBX1 was in the opposite situation. The difference of RBX2 expression between LUSC and adjacent tissues was much more obvious than that of RBX1 (**Figure 1B**). Meanwhile, the overall expression level of RBX2 was higher than that of RBX1 in pan-cancer (**Figure 1C**). We also analyzed that RBX1 and RBX2 are the two genes with significant positive correlation (Correlation coefficient = 0.49, **Figure 1D**).

**Figure 2:**
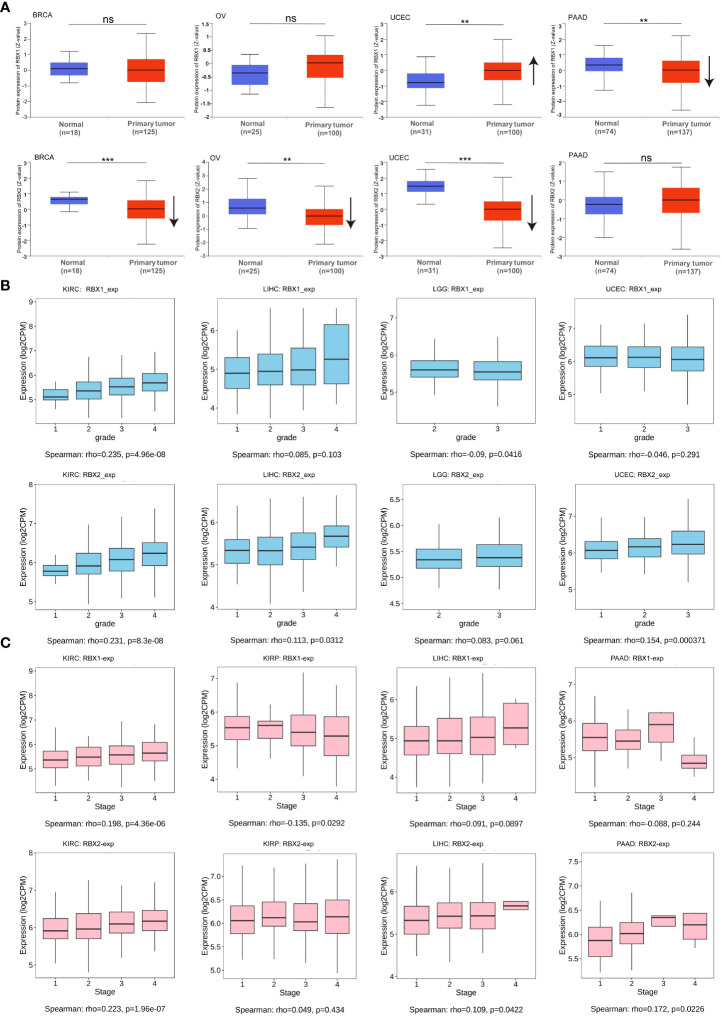
RBX1/2 expression based on tumor types and individual pathological grades and stages. **(A)** RBX-proteins expression in BRCA, OV, UCEC and PAAD. ns is considered as no statistical difference, **P < 0.01, ***P < 0.001. Up or down arrow represented the expression of tumor samples more or less than the corresponding normal samples, respectively. **(B)** The expression levels of RBX1/2 were analyzed by tumor pathological grades (grade1, grade2, grade3, grade4) of KIRC, LIHC, LGG and UCEC. P value less than 0.05 is considered as difference. **(C)** The expression levels of RBX1/2 were analyzed by tumor pathological stages (stage I, stage II, stage III, and stage IV) of KIRC, KIRP, LIHC and PAAD. P value less than 0.05 is considered as difference.

We investigated the RBX-proteins expression levels in BRCA, OV, UCEC and PAAD (**Figure 2A**). The results showed RBX2 expression in BRCA and OV was lower than in normal tissues, while RBX1 expression had no significant difference on the above tumors. Moreover, there was no difference on RBX2 expression in PAAD, however, RBX1 expression was higher in matched normal tissues. Another interesting phenomenon that RBX-proteins expression in UCEC was exact opposite and statistically significant was also illustrated. We showed RBX1/2 expression with pathological grades of KIRC, LIHC, LGG and UCEC using TISIDB database (**Figure 2B**), revealing that there were no differences in the association between RBX1 expression and clinical grades in LIHC and UCEC, whereas RBX2 expression has statistical significance in association with pathological grade of KIRC, LIHC and UCEC. We also observed the significant correlation between RBX1/2 expression and the pathological stages of several cancers including KIRC, KIRP, LIHC and PAAD (**Figure 2C**). The expression of RBX1 was not related to the stage of LIHC and PAAD, while RBX2 was in the opposite situation. Moreover, the association with RBX1/2 expression and KIRP stages was completely opposite, RBX1 was significantly correlated with the stages of KIRP. In conclusion, different expression patterns of RBX1/2 in various cancer types may lead to different characterization of tumors.

### Prognostic value of RBX1/2 across cancer types

**Figure 3:**
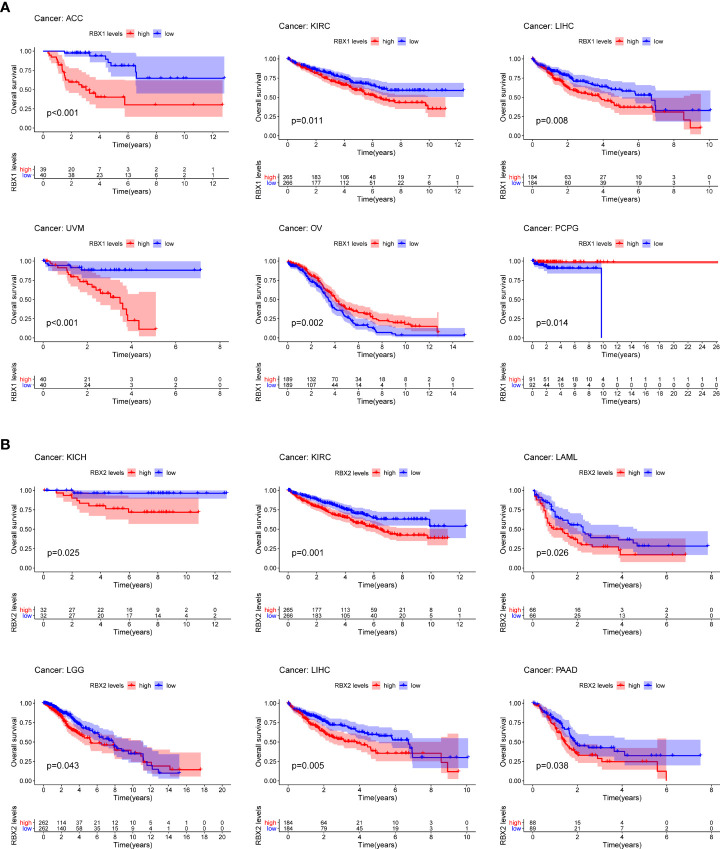
Kaplan-Meier survival curves comparison of high and low expression of Ring finger family gene in pan-cancer. **(A)** OS survival curves of RBX1 in different cancers: ACC, p<0.001; KIRC, p=0.011; LIHC, p=0.008; UVM, p<0.001; OV, p=0.002; PCPG, p=0.014. **(B)** OS survival curves of RBX2 in different cancers: KICH, p=0.025; KIRC, p=0.001; LAML, p=0.026; LGG, p=0.043; LIHC, p=0.005; PAAD, p=0.038.

The survival analysis of TCGA database presented a correlation between Ring finger family gene expression and prognosis in several cancers, showing that higher RBX1 expression was associated with poor OS in ACC (P<0.001), KIRC (P=0.011), LIHC (P=0.008), and UVM (P<0.001) (**Figure 3A**), whereas higher RBX2 expression was linked to poor prognosis in KICH (P=0.025), KIRC (P=0.001), LAML (P=0.026), LGG (P=0.043), LIHC (P=0.005) and PAAD (P=0.038) (**Figure 3B**). Interestingly, RBX1 had a protective role in OV (P=0.002), PCPG (P=0.014), suggesting RBX1 may exert tumor suppressor effect in OV and PCPG (**Figure 3A**).

**Figure 4:**
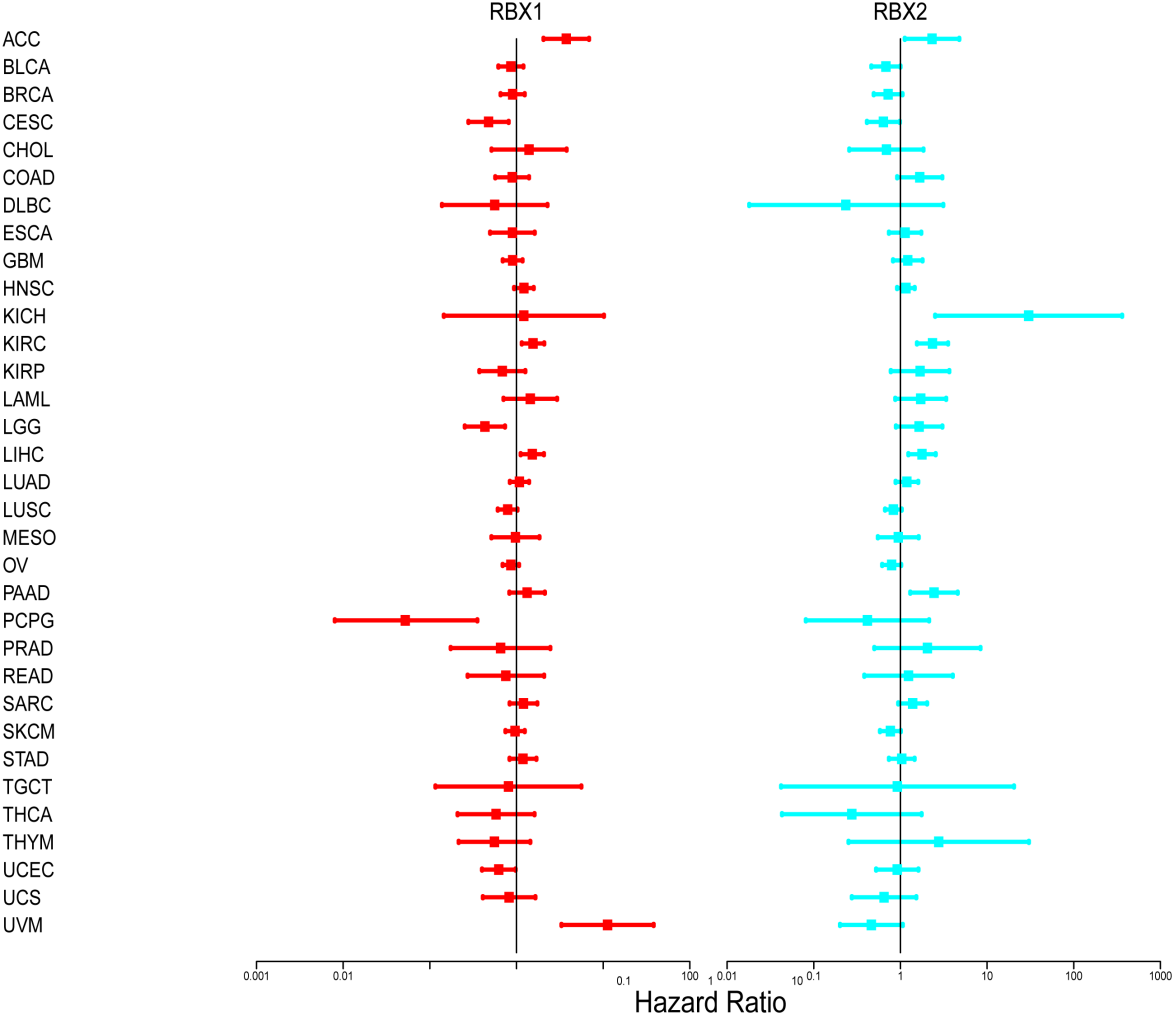
Association of RBX1/2 gene expression with patient’s overall survival for different cancer types. The forest plots with the hazard ratios and 95% confidence intervals for overall survival for different cancer types showing the survival advantage and disadvantage with the increased gene expression of RBX1/2. The univariate Cox proportional hazard regression models were used for the association tests.

We further investigated prognosis risk of the Ring finger family genes in pan-cancer by COX analysis (**Figure 4**). Our results indicated that RBX1 played a detrimental role in ACC, KIRC, LIHC and UVM (HR*>*1, *P<*0.05). On the other hand, RBX1 had a protective role in LGG, PCPG and CESC (HR<1, P<0.05). RBX2 acted as a detrimental prognostic factor in ACC, KICH, KIRC, LIHC and PAAD (HR*>*1, *P<*0.05). In contrast, RBX2 was a protective prognostic factor in CESC (HR<1, P<0.05). We have enumerated three tumors of the highest incidence (breast, colorectal and lung cancer) to perform comprehensive prognosis analysis with RBX1/2 expression by the PrognoScan database (35) (**Table 1**). RBX1 and RBX2 were the high-risk genes in breast cancer (RFS). Notably, RBX2 acted as a detrimental prognostic factor in colorectal cancer (OS, DFS) and lung cancer (OS, RFS). However, RBX1 had no significant relation with the prognosis in above cancers. The difference between RBX1 and RBX2 may lead to different tumor outcomes.

**Table 1:** Ring finger family gene expression was related to the prognosis of different cancers in PrognoScan.

Gene	Dataset	Cancer type	Endpoint	Number	COX P-value	HR	95% CI (low-high)
RBX1	GSE1456	Breast cancer	RFS	159	0.026281	1.01	1.13-6.70
RBX1	GSE7378	Breast cancer	DFS	54	0.602293	0.33	0.40-4.92
RBX1	GSE17537	Colorectal cancer	OS	55	0.992477	-0.01	0.26-3.73
RBX1	GSE17536	Colorectal cancer	DFS	145	0.707538	0.19	0.44-3.36
RBX1	GSE13213	Lung cancer	OS	117	0.051215	0.76	1.00-4.63
RBX1	GSE31210	Lung cancer	RFS	204	0.082506	0.81	0.90-5.58
RBX2	GSE1456	Breast cancer	RFS	159	0.002736	1.32	1.58-8.81
RBX2	GSE7378	Breast cancer	DFS	54	0.046315	-0.96	0.15-0.98
RBX2	GSE17537	Colorectal cancer	OS	55	0.041411	1.20	1.05-10.58
RBX2	GSE17536	Colorectal cancer	DFS	145	0.043114	1.07	1.03-8.20
RBX2	GSE13213	Lung cancer	OS	117	0.000608	1.12	1.62-5.83
RBX2	GSE31210	Lung cancer	RFS	204	0.000007	1.64	2.52-10.58

RFS, relapse free survival; DFS,Disease Free Survival; OS, overall survival; HR, hazard ratio; CI, Confidence Interval.

### RBX1/2 CNV profile in pan-cancer based on GSCA analysis

**Figure 5:**
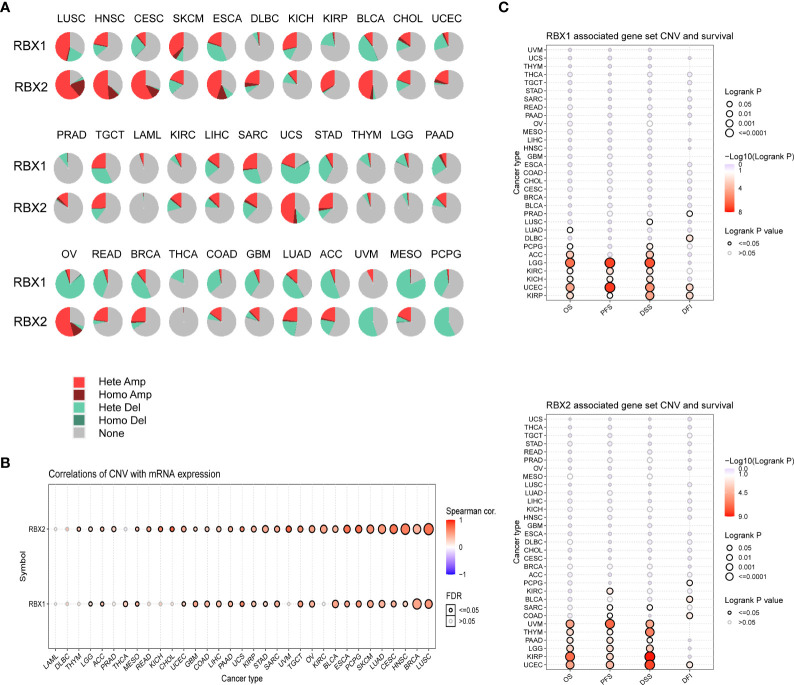
The CNV landscape of RBX1/2 in pan-cancer based on GSCA. **(A)** The deletion/amplification of heterozygous/homozygous CNV for RBX1/2 in pan-cancer. **(B)** Correlation between CNV and RBX1/2 mRNA expression in various cancers. **(C)** The profile of survival between RBX1/2 associated gene set CNV groups in various cancer types. CNV, copy number variation; GSCA, Gene Set Cancer Analysis; Hete Amp, Heterozygous Amplification; Homo Amp, Homozygous Amplification; Hete Del, Heterozygous Deletion; Homo Del, Homozygous Deletion; OS, overall survival; PFS, progression-free survival; DSS, disease specific survival; DFI, disease-free interval.

We summarized RBX1/2 CNV landscape in 33 cancer types by using the GSCA database, respectively (**Figure 5**). The highest heterozygous amplification ratio (45.71%) for RBX1 was found in LUSC, whereas the heterozygous amplification ratio of RBX2 presented a higher level of state in several cancers (>50%) including CESC, HNSC, LUSC and OV. Furthermore, a relatively higher heterozygous deletion ratio (>50%) for RBX1 was found in MESO, OV and UCS. However, RBX2 showed a heterozygous deletion ratio of more than 50% only in PCPG. The homozygous amplification of RBX2, had a significant proportion in some specific cancers containing CESC, ESCA, HNSC, LUSC and OV, for example, RBX2 homozygous amplification in LUSC was accounted for about 20% (**Figure 5A**). We also explored the association between RBX1/2 CNV and their mRNA expression (**Figure 5B**). Except for CHOL, DLBC, KICH, KIRC, LAML, PRAD, READ, THYM and UVM, the rest 24 cancer types were statistically significant for the correlation between RBX1 CNV and its mRNA expression. In addition to DLBC, LAML and THCA, RBX2 CNV had also a statistical significance with its mRNA expression in most cancers (**Figure 5B**). Subsequently, the profile of survival between the two members associated gene set CNV groups in the selected cancers was also summarized. The results suggested that wide type RBX1 had all statistical significance on OS, PFS, DFS and DFI in UCEC and KIRP. However, wide type RBX2 had all statistical significance on above four survival indicators only in UCEC (**Figure 5C**).

### RBX1/2 expression is related to immune subtypes in cancers

**Figure 6:**
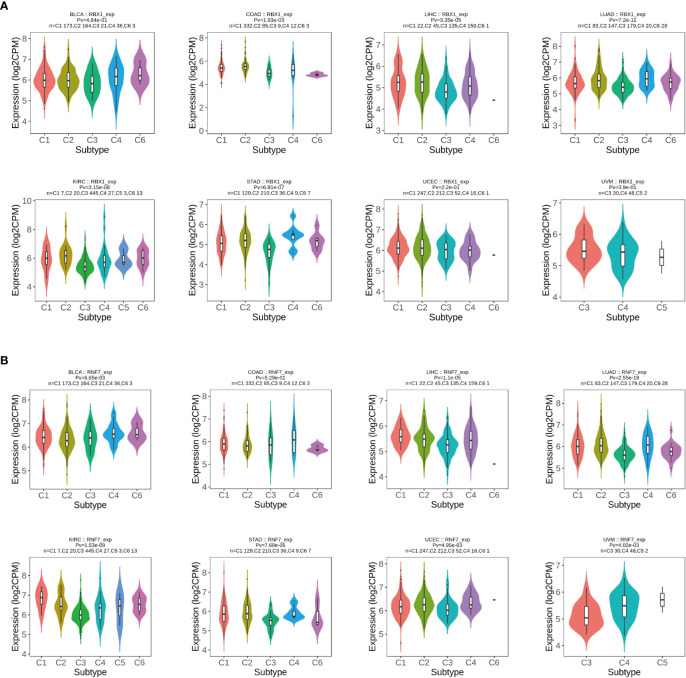
The relationship between RBX1/2 expression and pan-cancer immune subtypes. **(A)** Correlation of RBX1 expression and immune subtypes in BRCA, COAD, LIHC, LUAD, KIRC, STAD, UCEC and UVM. **(B)** Correlation of RBX2 expression and immune subtypes in BRCA, COAD, LIHC, LUAD, KIRC, STAD, UCEC and UVM. P value less than 0.05 is considered as difference.

Previous studies determined that RBX1 and RBX2 were involved in immunomodulatory processes (19, 36), therefore, we compared the relationships between RBX1/2 expression and immune subtypes through the TISIDB database (**Figure 6**). Immune subtypes were classified into six types, including C1 (wound healing), C2 (IFN-gamma dominant), C3 (inflammatory), C4 (lymphocyte depleted), C5 (immunologically quiet) and C6 (TGF-b dominant). Our analyses showed that RBX1 expression in the immune subtypes of BLCA, UCEC and UVM had no statistical significance, while RBX2 expression in above three cancers was closely related with those immune subtypes. Conversely, RBX2 expression had no correlation with the COAD immune subtypes. Of interest, taking KIRC as the example, RBX1 showed high expression in C2 and C6 types, however, RBX2 expression on C1 immune subtype was the highest in KIRC. Furthermore, we investigated the association with RBX1/2 expression and immune subtypes in the TCGA pan-cancer data, illustrating that the expression of RBX1 was lowest in the C3 immune subtype, while RBX2 was lowest in the C5 immune subtype (**Supplementary Figure 3**). Based on the above results, we concluded that RBX1/2 expression differs in immune subtypes of various tumor cancers.

### Association between RBX1/2 mRNA expression and immune infiltration in pan-cancer

**Figure 7:**
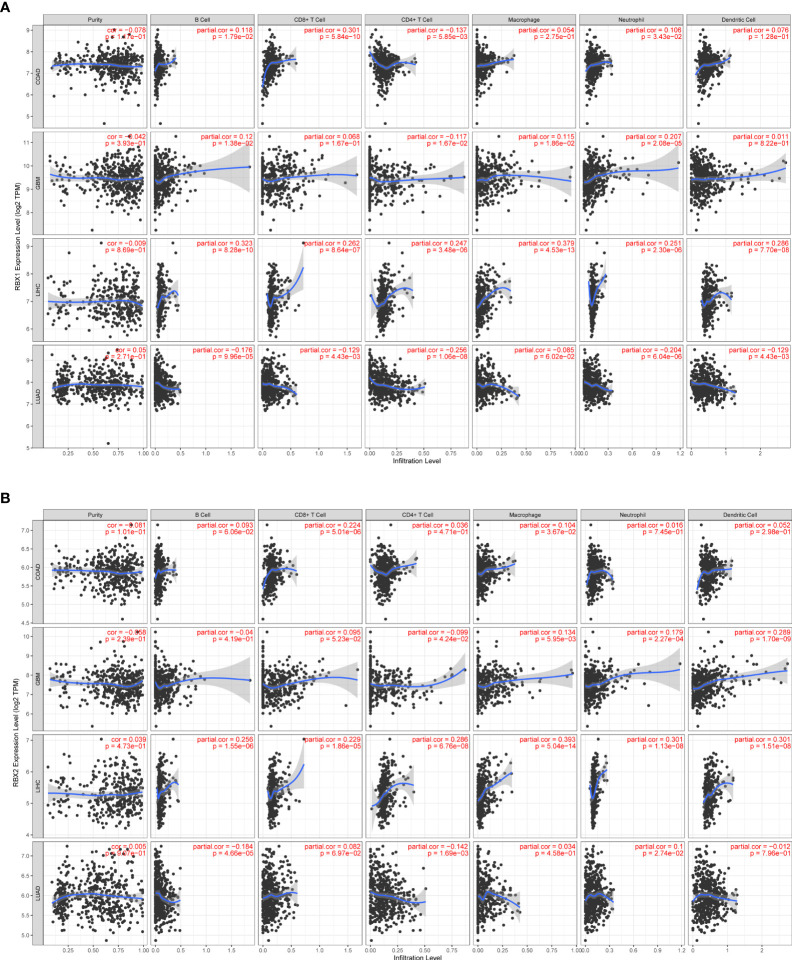
Correlation of RBX1/2 expression with immune infiltration level in COAD, GBM, LIHC and LUAD. **(A)** RBX1 expression is related with the level of immune infiltration in the above four cancers. **(B)** RBX2 expression is related with the level of immune infiltration in the above four cancers. P value less than 0.05 is considered as difference.

**Figure 8:**
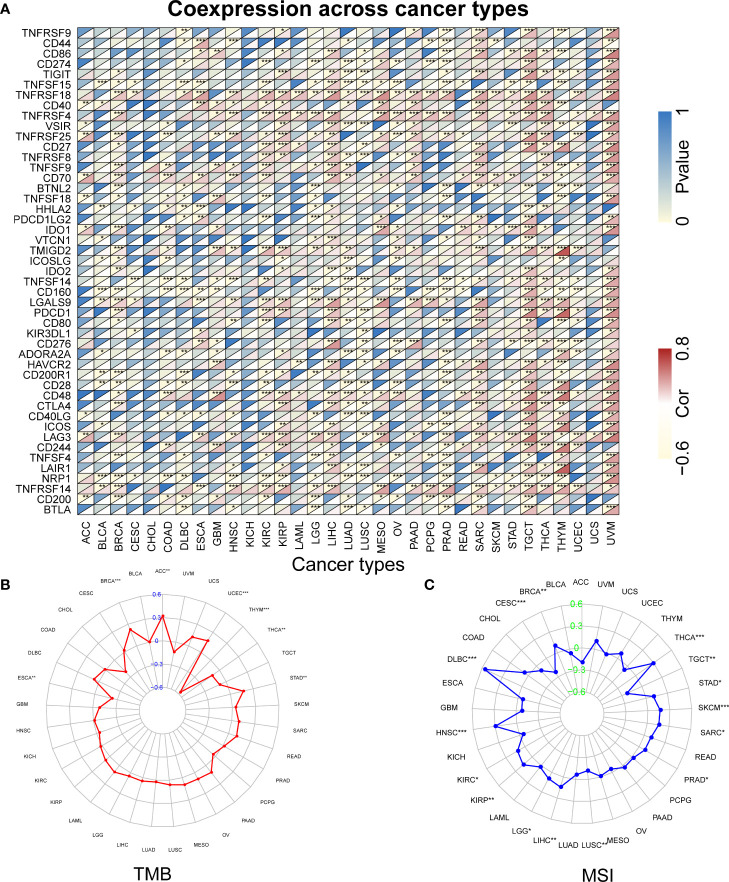
The relationship between RBX1 and immune checkpoints, TMB and MSI based on TCGA database. **(A)** Heatmap illustrating the relationship between RBX1 and known immune checkpoints. The top left triangle represents the P-value, and the bottom right triangle represents the correlation coefficient. Correlation between RBX1 and TMB **(B)** and MSI **(C)**. *P < 0.05, **P < 0.01, and ***P < 0.001.

**Figure 9:**
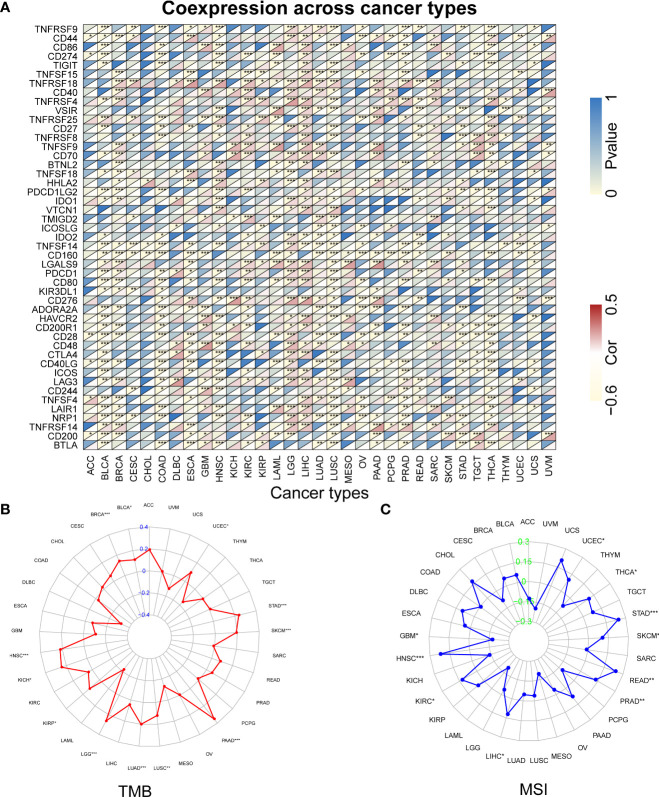
The relationship between RBX2 and immune checkpoints, TMB and MSI based on TCGA database. **(A)** Heatmap illustrating the relationship between RBX2 and known immune checkpoints. The top left triangle represents the P-value, and the bottom right triangle represents the correlation coefficient. Correlation between RBX2 and TMB **(B)** and MSI **(C)**. *P < 0.05, **P < 0.01, and ***P < 0.001.

Studies indicated that RBX1 expression are associated with the immune suppressive function of Treg cells, and T-cell deficiency, and RBX2 could trigger a series of immune responses, suggesting they may play important roles in regulating immune cells (37, 38). We found a strong correlation between RBX1/2 expression and the levels of immune infiltration in COAD, GBM, LIHC and LUAD by analysis of the TIMER database (**Figure 7**). The expression of RBX1 was in connection with the infiltration of B cell, CD4^+^ T cells and neutrophils in above four cancers (**Figure 7A**). With regard to RBX2, the infiltration of CD8^+^ T cells and macrophages have a positive correlation with RBX2 in COAD and LIHC (**Figure 7B**). We also conducted the co-expression analysis to further explore the association between RBX1/2 expression and immune checkpoints in pan-cancer using the TCGA database. As shown in **Figure 8A**, RBX1 was positively correlated with these immune markers in SARC, TCGT and UVM, whereas the positive association between RBX2 mRNA and immune checkpoints existed in LGG and LIHC (**Figure 9A**). Interestingly, we found that RBX1 was positively correlated with the expression levels of PD1 (PDCD1) and CTLA-4 in BRCA, KIRP, LIHC, SARC, TCGT, THCA and UVM (**Figure 8A**). RBX2 had a closely tie with the expression level of PD-L1 (CD274) in BLCA, COAD, HNSC, KIRC, LAML, LIHC, OV, PCPG, PRAD, SKCM, TGCT and THCA (**Figure 9A**). These results indicated that RBX1/2 might regulate different immune response in various cancer types.

### RBX1/2 expression is related to tumor mutational burden, microsatellite instability and tumor microenvironment

Further analysis found that RBX1 expression was positively correlated with TMB in ACC, BRCA, STAD and UCEC, but negatively correlated with ESCA, THCA and THYM, as seen in **Figure 8B**. However, RBX2 expression had no relation with TMB in ACC, ESCA, THCA and THYM (**Figure 9B**). We also found that the RBX1 had a positive association with MSI in BRCA, DLBC, HNSC, KIRC, KIRP, LGG, LIHC, PRAD, SARC, SKCM, STAD and THCA, but had a negative association with CESC, LUSC and TGCT, as seen in **Figure 8C**. Similarly, correlation analysis between RBX2 expression and MSI was also performed (**Figure 9C**). In HNSC, KIRC, LIHC, PRAD, READ, SKCM, STAD, THCA and UCEC, RBX2 expression was positively related to MSI, whereas the expression of RBX2 has a negative relationship with GBM (**Figure 9C**).

To obtain a more comprehensive analysis of the relationship between Ring finger family and immune components, we applied the estimate algorithm to evaluate the stromal and immune scores in 33 cancer types. RBX1/2 existed statistically significance in stromal, immune, and estimate scores (**Supplementary Figures 4A-C**). Besides, they had a significantly positive or negative correlation with DNAss, RNAss and tumor purity in pan-cancer (**Supplementary Figures 4D-F**). These results suggested RBX1/2 may be involved in different immune processes in various cancer types.

## Discussion

Previous studies have systematically provided a comprehensive overview on the alterations of SCF E3 ubiquitin ligases in the pathogenesis and development of cancers (39, 40). RBX1/2 were overexpressed in a number of primary cancer tissues, including carcinoma of lung, liver, breast, colon, and renal. Sun Y et al. has demonstrated that inactivation of either RBX1 or RBX2 inhibits carcinogenesis *via* various mechanisms, including apoptosis and senescence (17, 41, 42). However, two other studies found that only RBX2 overexpression was correlated with the poor prognosis in lung cancer (21); as well as high RBX1 expression was related to poor survival only in KIRC patients and high RBX2 expression had a close relation with poor prognosis in all three types of RCC (22, 43). At present, the comparison of Ring finger family in the same cancer is rare. The underlying mechanisms by which they contribute to different outcomes in cancer patients remains largely unknown. Therefore, we focused on their differences in mRNA level, protein level, pathological parameters, prognosis and etc. by the pan-cancer analysis in this study **Supplementary Table 1**. Our result showed that RBX1/2 reflected their characteristics respectively in the observation indicators mentioned, for example, RBX2 expression is more differentially expressed than RBX1 in LUSC, which may be one of the reasons that only RBX2 expression is associated with lung cancer prognosis.

Accumulating evidence suggests that the E3s dysfunction can contribute to adverse immune response (44–46). Previously, several studies have observed that RBX1 can promote ubiquitin degradation of HBx-induced PD-L1 protein in HCC cells (47). Meanwhile, RBX2-dependent neddylation played a significant role in the regulation of T-cell responses (38). Thus, there is a dire need for exploring the relationship of RBX1/2 expression and immune components. Using bioinformatics methods, we elucidated the immunological role of the Ring finger family across cancers and provided in first time the gene expression and genetic alteration of RBX1/2 in the regulation of different immune components including their association with PD-L1 expression. This result showed RBX1/2 may be attractive biomarkers of immunotherapy efficacy.

We investigated and integrated information based on bioinformatics and public databases, however, there were still some limitations in the present study. First, whether the Ring finger family is harmful or beneficial remains contradictory because of some conflicting findings from different databases. Second, despite the finding that they were closely associated with immune infiltration and prognosis, we were unable to determine whether these two molecules affected patient survival through immune infiltration. Finally, whether differences in RBX-proteins are a decisive factor in the stability of the SCF complex in pan-cancer needs to be further clarified.

In summary, our results revealed that the important role of Ring finger members in the SCF complex, and the expression profile of RBX1/2 in pan-cancer. Moreover, strong correlations between RBX1/2 and disease prognosis and immune components were proved in the present study. Clinical immune markers, such as PD-1, CTLA-4 and PD-L1, have been confirmed to be closely associated with Ring finger family in a variety of cancers. These findings may provide insights for further investigation of the Ring finger family genes as potential targets in pan-cancer.

## Data availability statement

The data that support the findings of our study are openly available from the TCGA, UALCAN, TISIDB, PrognoScan, GSCALite and Timer database at (https://tcgadata.nci.nih.gov/tcga/,http://ualcan.path.uab.edu/, http://cis.hku.hk/TISIDB/index.php, http://www.abren.net/PrognoScan/, http://bioinfo.life.hust.edu.cn/web/GSCALite/, https://cistrome.shinyapps.io/timer/).

## Author contributions

All authors read and approved the final manuscript. HA designed the overall study and revised the paper. TH and JL drafted the manuscript and performed the data analysis. BS, XL and SL participated in the data collection. All authors contributed to the article and approved the submitted version

## Funding

This project is supported by the grand “Peking Medical and Health Foundation.” Nr.: F3142C

## Acknowledgments

We thank the databases of the TCGA, UALCAN, TISIDB, PrognoScan, GSCALite, Timer for the availability of the data.

## Conflict of interest

The authors declare that the research was conducted in the absence of any commercial or financial relationships that could be construed as a potential conflict of interest.

## Publisher’s note

All claims expressed in this article are solely those of the authors and do not necessarily represent those of their affiliated organizations, or those of the publisher, the editors and the reviewers. Any product that may be evaluated in this article, or claim that may be made by its manufacturer, is not guaranteed or endorsed by the publisher.

## Supplementary material

Supplementary Table 1 The summarization of alterations of RBX1 and RBX2 in all types of cancers. These statistical data are mainly from online databases and R package processed.

## Glossary

**Table:**

RBX1/ROC1	ring box protein 1
RBX2/SAG/RNF7	rign box protein 2
CUL1	cullin1
OS	overall Survival
DFS	disease Free Survival
DSS	disease specific survival
RFS	relapse free survival
DMFS	distant Metastasis-Free Survival
PFS	progression-Free-Survival
DFI	disease free interval
CNV	copy number variation
TMB	tumor mutational burden
MSI	microsatellite instability
ACC	adrenocortical carcinoma
BLCA	bladder urothelial carcinoma
BRCA	breast invasive carcinoma
CESC	cervical squamous cell carcinoma and endocervical adenocarcinoma
CHOL	cholangiocarcinoma
COAD	colon adenocarcinoma
DLBC	lymphoid neoplasm diffuse large b-cell lymphoma
ESCA	esophageal carcinoma
GBM	glioblastoma multiforme
HNSC	head and neck squamous cell carcinoma
KICH	kidney chromophobe
KIRC	kidney renal clear cell carcinoma
KIRP	kidney renal papillary cell carcinoma
LAML	acute myeloid leukemia
LGG	brain lower grade glioma
LIHC	liver hepatocellular carcinoma
LUAD	lung adenocarcinoma
LUSC	lung squamous cell carcinoma
MESO	mesothelioma
OV	ovarian serous cystadenocarcinoma
PAAD	pancreatic adenocarcinoma
PCPG	pheochromocytoma and paraganglioma
PRAD	prostate adenocarcinoma
READ	rectum adenocarcinoma
SARC	sarcoma
SKCM	skin cutaneous melanoma
STAD	stomach adenocarcinoma
TGCT	testicular germ cell tumors
THYM	thymoma
THCA	thyroid carcinoma
UCS	uterine carcinosarcoma
UCEC	uterine corpus endometrial carcinoma
UVM	uveal melanoma
