# Supplementary material for: An integrative pan-cancer analysis revealing the difference in small ring finger family of SCF E3 ubiquitin ligases
Source: Front Immunol. 2022 Aug 18;13:968777. doi: 10.3389/fimmu.2022.968777 (PMC9434121; doi:10.3389/fimmu.2022.968777)
Supplement: Supplementary file 1 [file DataSheet_1.docx]

Supplementary Material

**Supplementary Figures**


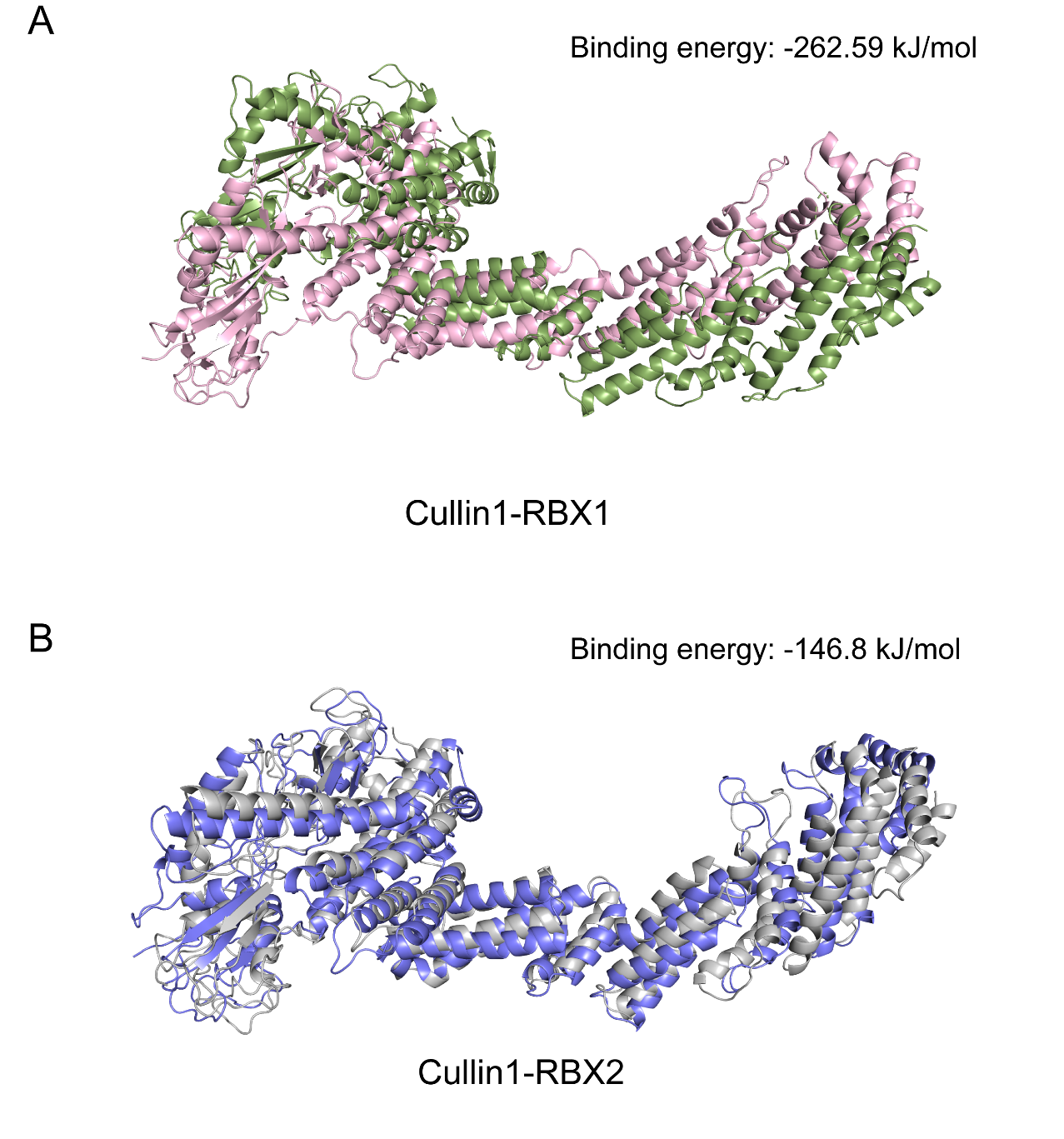
 **Figure 1:**


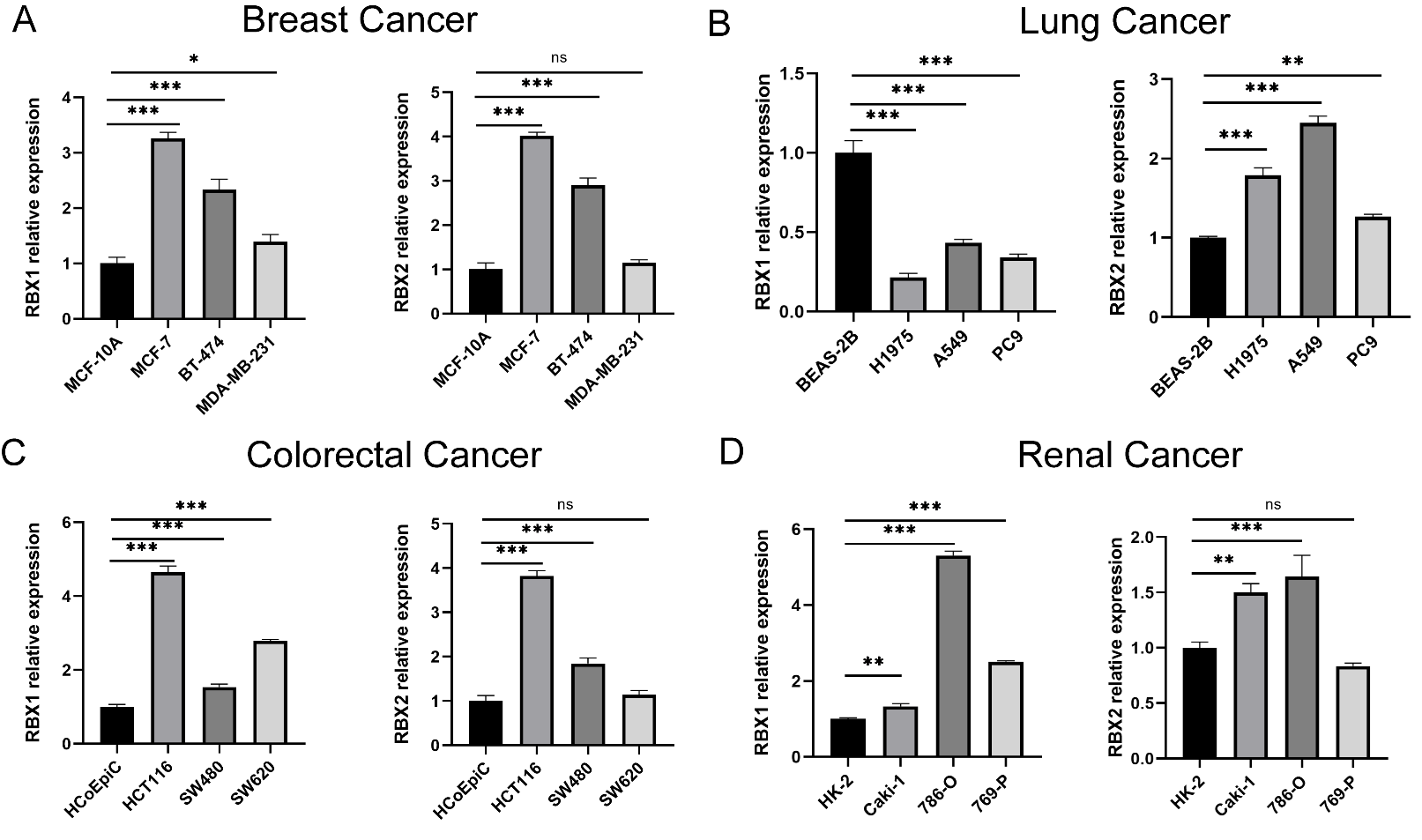
**Figure 2:**

**Figure 3:**


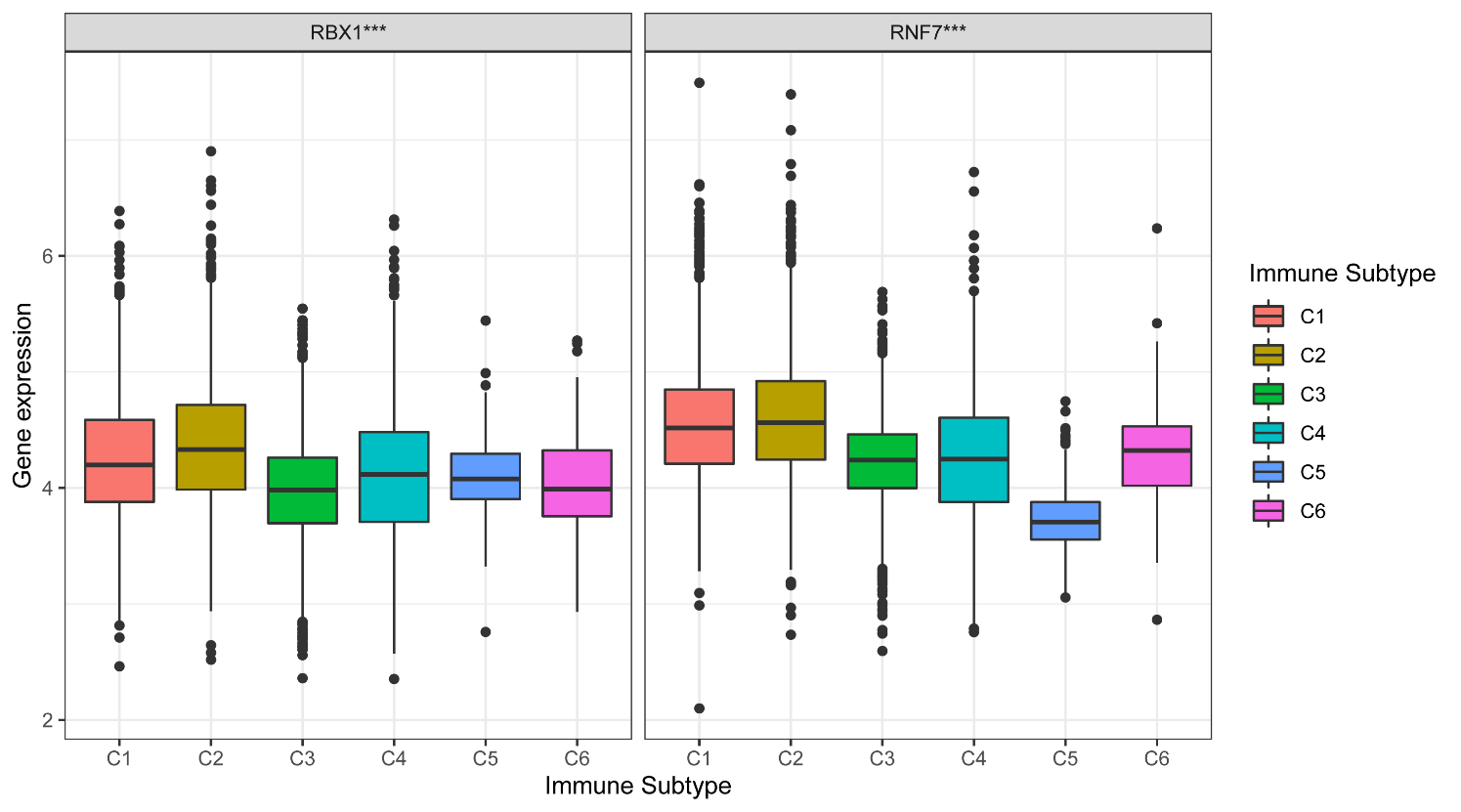


**
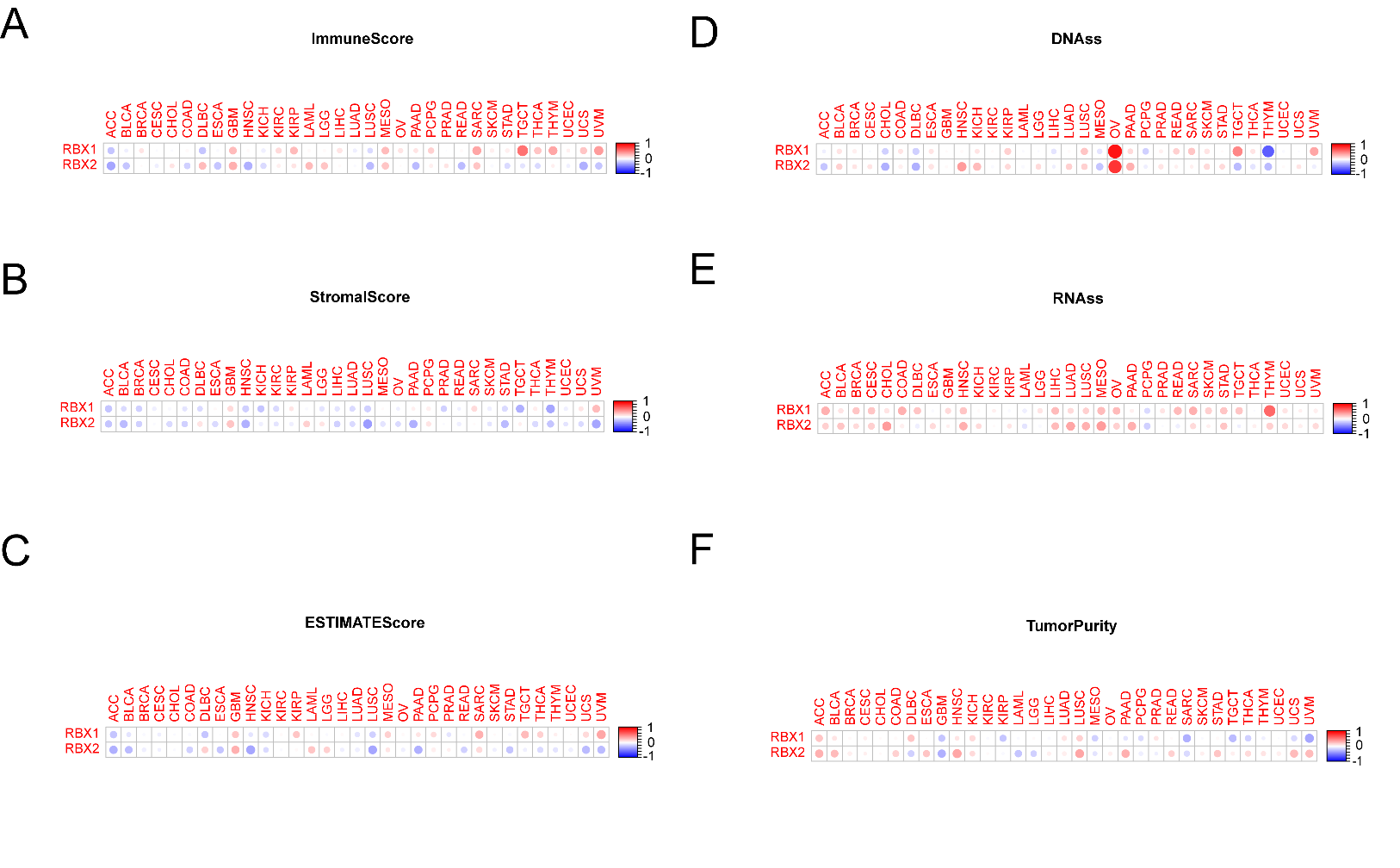
Figure 4:**

**Supplementary Figure legends**

**Fig. 1.** Overall structure of CUL-RBX1 (A) and CUL1-RBX2 complex (B). The original conformation of CUL1-RBX1 is colored in pink and the last conformation is colored in green. Whereas the initial conformation of CUL1-RBX2 is colored in gray and the ultimate conformation is colored in blue.

**Fig.2.** Relative RBX family members’ mRNA levels in specific four types of cancer. (A) Breast Cancer, (B) Lung Cancer, (C) Colorectal Cancer, and (D) Renal Cancer. The experiments were repeated three times. ∗P< 0.05, ∗∗P< 0.01, and ∗∗∗P< 0.001.

**Fig. 3.** Correlation between Ring finger family gene expression and immune subtypes in all 33 cancer types.

**Fig. 4.** Association between Ring finger family gene expression and tumor micro-environment factors and stemness score in pan-cancer. The Ring finger family gene associated immune score (A), stromal score (B), estimate score (C), DNAss (D), RNAss (E) and tumor purity (F) are illustrated.
