# Supplementary material for: An integrative pan-cancer analysis revealing the difference in small ring finger family of SCF E3 ubiquitin ligases
Source: Front Immunol. 2022 Aug 18;13:968777. doi: 10.3389/fimmu.2022.968777 (PMC9434121; doi:10.3389/fimmu.2022.968777)
Supplement: Supplementary Table 1 — The summarization of alterations of RBX1 and RBX2 in all types of cancers. These statistical data are mainly from online databases and R package processed. [file Table_1.docx]

Supplementary Table 1. The summarization of alterations of RBX1 and RBX2

in all types of cancers.

| **Characteristic** |  | RBX1 | RBX2 |
| --- | --- | --- | --- |
| **Gene expression** | High | BLCA, BRCA, CHOL, COAD, ESCA, GBM, HNSC, KIRC, LIHC, LUSC, PRAD, | BLCA, BRCA, CHOL, ESCA, GBM, HNSC, KIRC, KIRP, LIHC, LUAD, LUSC, PRAD, |
|  | Low | KICH | COAD, KICH, READ |
| **Protein expression** | High | UCEC, HNSC, LIHC |  |
|  | Low | LUAD, PAAD, GBM | BRCA, OV, KIRC, UCEC, LUAD, HNSC, |
| **Overall-survival** | Better | OV, PCPG |  |
|  | Worse | ACC, LIHC, KIRC, UVM | KICH, KIRC, LAML, LGG, LIHC, PAAD |
| **Cox analysis** | HR>1 | ACC, KIRC, LIHC, UVM | ACC, KICH, KIRC, LIHC, PAAD |
|  | HR<1 | LGG, PCPG, CESC | CESC |
| **Stage** |  | ACC, KIRC, KIRP, LIHC, TGCT | ACC, HNSC, KIRC, LIHC, PAAD |
| **TMB** |  | ACC, BRCA, ESCA, STA D, THCA, THYM, UCEC | BLCA, BRCA, HNSC, KICH, KIRP, LGG, LUAD, LUSC, PAAD, SKCM, STAD, UCEC |
| **MSI** |  | BRCA, CESC, DLBC, HN SC, KIRC, KIRP, LGG, LI HC,LUSC, SARC, SKCM, STAD, TGCT, THCA | GBM, HNSC, KIRC, LIHC, PRAD, READ, SKCM, STAD, THCA, UCEC |
| **With PD-L1**  **association** | Positive | LGG, MESO, TGCT, UVM | LIHC, SKCM |
|  | Negative | DLBC, KIRC, LUAD,  LUSC, OV, PCPG, PRAD, | ACC, BLCA, COAD, HNSC, KIRC, LAML, OV, PCPG, PRAD, TGCT, THCA |

These statistical data are mainly from online databases and R package processed.

1
